# A multi-functional chemosensor for highly selective ratiometric fluorescent detection of silver(I) ion and dual turn-on fluorescent and colorimetric detection of sulfide

**DOI:** 10.1098/rsos.180293

**Published:** 2018-06-13

**Authors:** Ji Hye Kang, Ju Byeong Chae, Cheal Kim

**Affiliations:** Department of Fine Chemistry, Seoul National University of Science and Technology, Seoul 139-741, Korea

**Keywords:** fluorescent, colorimetric, chemosensor, silver, sulfide, theoretical calculations

## Abstract

A multi-functional chemosensor **1** as silver and sulfide detector was synthesized by the combination of octopamine and 4-dimethylaminocinnamaldehyde. Sensor **1** exhibited a ratiometric fluorescence emission for Ag^+^ from blue to sky. The binding mode of **1** and Ag^+^ turned out to be a 1 : 1 ratio as determined using Job plot and electrospray ionization (ESI) mass spectral analyses. The sensing mechanism of **1** with silver ion was unravelled by ^1^H NMR titrations and theoretical calculations. Sensor **1** also discerned sulfide by enhancing fluorescence intensity and changing colour from yellow to colourless in aqueous solution. The sensing properties of **1** toward S^2−^ were investigated by using ESI-mass analysis, Job plot and ^1^H NMR titrations. Moreover, **1** could be used as a detector for sulfide in a wide pH range.

## 1. Introduction

To date, many researchers have studied many analytical methods for the detection of various analytes of metal ions and anions such as atomic absorption spectroscopy, surface-enhanced Raman scattering, ion-selective electrodes and inductively coupled plasma mass spectrometry [1–6]. However, these methods have required complicated procedures, operator and great expense [7–11]. In contrast, a chemosensor using fluorescent and colorimetric responses has many advantages such as rapid signalling time, high selectivity and operational simplicity [12–18]. Therefore, it is worthwhile to design chemosensors for the detection of cations and anions.

Silver(I) is one of frequently used metals in the industrial field like imaging, electronics, pharmacy and photography as well as in real life like jewellery, table cutlery and filters for water purifiers [19,20]. However, superabundance of silver can cause a strong poison to organisms and environments [21,22]. For example, Ag^+^ which has a strong interaction with amino and sulfhydryl groups can easily form hazardous complexes with nucleic acids, amino acids and so on [23,24]. Therefore, it is an acute environmental pollutant owing to a large consumption of silver [25–27]. These facts led us to consider that it is of importance to determine silver(I) in the surrounding environment.

Sulfide in organisms has been known as an endogenous signalling molecule, which functions in various biological reactions [28,29]. For instance, S^2−^ can inhibit vascular smooth muscle cell proliferation, regulate apoptosis and interfere with insulin signalling [30,31]. Though sulfide is generated endogenously from l-cysteine, abnormal concentrations of sulfide in bio-systems can lead to serious trouble such as hypertension, diabetes, liver cirrhosis and Down's syndrome [32–35]. Therefore, development of a sulfide detector has been receiving substantial attention [36,37].

Octopamine is associated with regulations of blood pressure and weight loss, and used as a sympathomimetic drug [38,39]. Thus, it is considered as a bio-friendly molecule. Cinnamaldehyde moiety is known to be a good chromophore and fluorophore which emits in the range of 400–550 nm [40–44]. Thus, we expected that a sensor containing both an octopamine moiety and a cinnamaldehyde one might act as a unique chemosensor.

Herein, we present a novel chemosensor **1** for targeting silver(I) and sulfide with various responses. Sensor **1** could detect Ag^+^ with red-shifted fluorescent emission from 449 (blue) to 488 nm (sky). The binding structure and mechanism of **1** with Ag^+^ were explained via Job plot and electrospray ionization (ESI) mass spectral analyses, ^1^H NMR titration and theoretical calculations. Moreover, **1** could be applied to sense S^2−^ with dual methods, which were the fluorescence turn-on and colour change from yellow to colourless. The sensing mechanism of **1** toward sulfide was described by using Job plot, ESI-mass analyses and ^1^H NMR titration.

## 2. Experimental

### 2.1. Materials and instrumentation

Sigma-Aldrich provided all the reagents of analytical and spectroscopic grade. Absorption spectra and emission spectra were recorded using Lambda L25 UV/Vis and LS45 fluorescence spectrometers (Perkin Elmer), respectively. A Varian spectrometer recorded ^1^H and ^13^C NMR spectra. ESI mass spectra were measured with a Thermo Finnigan ion trap instrument.

### 2.2. Synthesis of **1**

Octopamine (194 mg) and triethylamine (TEA, 140 µl) were dissolved in 5 ml of methanol (MeOH). Then, 4-dimethylaminocinnamaldehyde (215 mg) was added dropwise into the reaction solution. The reaction mixture was stirred additionally for 1 h until the precipitate appeared. An orange precipitate obtained was filtered, washed three times with ethyl acetate, and dried to obtain the pure orange solid. The yield: 77.6%. ^1^H NMR (400 MHz, DMF-*d_7_*, ppm, 25°C): *δ* 9.42 (s, 1H), 8.01 (d, *J* = 8 Hz, 1H), 7.00 (d, *J* = 16 Hz, 1H), 7.46 (d, *J* = 8 Hz, 2H), 6.81 (d, *J* = 8 Hz, 2H), 7.24 (d, *J* = 8 Hz, 2H), 6.76 (d, *J* = 8 Hz, 2H), 6.72 (m, 1H), 5.05 (s, 1H), 4.80 (t, *J* = 8 Hz, 1H), 3.75 (d, *J* = 12 Hz, 1H), 3.58 (t, *J* = 12 Hz, 1H); ^13^C NMR (100 MHz, DMSO-*d_6_*, ppm, 25°C): *δ* = 164.5, 156.6, 151.3, 142.7, 135.1, 129.0, 127.7, 123.6, 123.4, 115.1, 112.4, 72.5, 69.3. ESI-MS: *m*/*z* calcd for C_19_H_22_N_2_O_2_ + H^+^, 311.18; found, 311.28.

### 2.3. Fluorescence titrations

For Ag^+^, 1 ml of dimethylformamide (DMF) was used to dissolve **1** (1.6 mg) and 3 µl of **1** (5 mM) was diluted with 2.997 ml of DMF to afford a 5 µM concentration. Amounts of 0.8–10.6 µl of a stock AgNO_3_ solution (10 mM) in DMF were transferred to the sensor **1** solution (5 µM) prepared above. After mixing them for 10 s, fluorescence spectra were recorded.

For S^2−^, 1 ml of DMF was used to dissolve **1** (1.6 mg) and 6 µl of **1** (5 mM) was diluted with 2.994 ml of bis-tris buffer (pH = 7.0, 10 mM) to make a 10 µM concentration. Amounts of 1.5–15.0 µl of a stock Na_2_S solution (200 mM) in bis-tris buffer were transferred to the sensor **1** solution (10 µM) prepared above. After mixing them for 10 s, fluorescence spectra were recorded.

### 2.4. UV–visible titrations

For Ag^+^, 1 ml of DMF was used to dissolve **1** (1.6 mg) and 3 µl of **1** (5 mM) was diluted with 2.997 ml of DMF to make a 5 µM concentration. Amounts of 6.0–78.0 µl of a stock AgNO_3_ solution (10 mM) in DMF were transferred to the sensor **1** solution (5 µM) prepared above. After mixing them for 10 s, UV–visible spectra were recorded.

For S^2−^, 1 ml of DMF was used to dissolve **1** (1.6 mg) and 6 µl of **1** (5 mM) was diluted with 2.994 ml of bis-tris buffer (pH = 7.0, 10 mM) to make a 10 µM concentration. Amounts of 1.5–18.0 µl of a stock Na_2_S solution (200 mM) in bis-tris buffer were transferred to the sensor **1** solution (10 µM) prepared above. After mixing them for 10 s, UV–visible spectra were recorded.

### 2.5. Job plots

For Ag^+^, 1 ml of DMF was used to dissolve **1** (1.6 mg) and 180 µl of **1** (5 mM) was diluted with 29.82 ml of DMF to make a 30 µM concentration. Amounts of 2.7–0.3 ml of the **1** solution were taken and transferred to fluorescence cells. 60 µl of a stock Ag^+^ solution (10 mM) in DMF was diluted with 19.97 ml DMF. 0.3–2.7 ml of the Ag^+^ solution was added to each **1** solution. After mixing them for 10 s, fluorescence spectra were recorded.

For S^2−^, 1 ml of DMF was used to dissolve **1** (1.6 mg) and 420 µl of **1** (5 mM) was diluted with 29.58 ml of bis-tris buffer (pH = 7.0, 10 mM) to make a final concentration of 70 µM. Amounts of 2.7–0.3 ml of the **1** solution were taken and transferred to UV cells. 7 µl of a stock S^2−^ solution (200 mM) in bis-tris buffer was diluted with 19.99 ml bis-tris buffer. 0.3–2.7 ml of the S^2−^ solution was added to each **1** solution. After mixing them for 10 s, UV–visible spectra were recorded.

### 2.6. Competitive experiments

For Ag^+^, MNO_3_ (0.1 mmol; M = Na, K) or M(NO_3_)_2_ (0.1 mmol; M = Cd, Zn, Fe, Cu, Hg, Ni, Co, Mg, Pb, Mn, Ca, Pd) or M(NO_3_)_3_ (0.1 mmol; M = Fe, Al, In, Ga, Cr) or AuCl_3_ (0.1 mmol) was separately dissolved in DMF (5 ml). 4.5 µl of each metal-ion solution (20 mM) was diluted with 2.988 ml of DMF to give 6 equiv of metal ions. 9.0 µl of a stock Ag^+^ solution (10 mM) in DMF was taken and added to each metal-ion solution prepared above. Then, 1 ml of DMF was used to dissolve **1** (1.6 mg) and 3 µl of **1** (5 mM) was taken and added to the mixed solution prepared above to make a 5 µM concentration. After mixing them for 10 s, fluorescence spectra were recorded.

For S^2−^, tetraethylammonium salts (Br^−^, CN^−^, Cl^−^, F^−^, I^−^; 1.0 mmol) or tetrabutylammonium salts (BzO^−^, OAc^−^, N_3_^−^, SCN^−^; 1.0 mmol) or sodium salts (Na_x_X) (*X* = H_2_PO_4_^−^, NO_2_^−^, SO_4_^2−^, OH^−^, HSO_3_^−^; 1.0 mmol) were separately dissolved in 5 ml of bis-tris buffer. 13.5 µl (or 16.5 µl for colorimetric response) of each anion solution (200 mM) was diluted with 2.967 ml of bis-tris buffer to give 90 equiv (or 110 equiv for colorimetric response) of anions. 13.5 µl (or 16.5 µl for colorimetric response) of 1.5–15.0 µl of a stock S^2−^ solution (200 mM) in bis-tris buffer was taken and added to each anion solution prepared above. Then, 1 ml of DMF was used to dissolve **1** (1.6 mg) and 6 µl of **1** (5 mM) was taken and added to the mixed solution prepared above to make a 10 µM concentration. After mixing them for 10 s, fluorescence and UV–visible spectra were recorded.

### 2.7. ^1^H NMR titrations

For Ag^+^, four NMR tubes of sensor **1** (0.01 mmol) dissolved in DMF-*d_7_* were prepared, and then three different equivalents (1, 2 and 4 equiv) of AgNO_3_ dissolved in DMF-*d_7_* were taken and added to each solution of sensor **1**. After shaking the solutions for 10 s, ^1^H NMR spectra were recorded.

For S^2−^, three NMR tubes of sensor **1** (0.01 mmol) dissolved in DMF-*d_7_*/D_2_O (6:1, v/v) were prepared, and then two different equivalents (1 and 2 equiv) of Na_2_S dissolved in D_2_O were taken and added to each solution of sensor **1**. After shaking the solutions for 10 s, ^1^H NMR spectra were recorded.

### 2.8. Theoretical calculations of **1** and **1**–Ag^+^

The density functional theory (DFT) and time-dependent DFT (TD-DFT) methods were used for all theoretical calculations with the Gaussian 03 program [45–47]. The main atoms were applied for 6-31G (d, p) basis set [48,49], and the silver element was applied for the LANL2DZ effective core potential (ECP) [50–52]. All geometries were optimized in the ground states (S_0_). All calculations were considered to the solvent effect of DMF using the Cossi and Barone conductor-like polarizable continuum model [53,54]. The transition energies for the minimized structures of **1** and **1**–Ag^+^ complex were determined by calculating the lowest 20 singlet–singlet transitions with TD-DFT calculations at the ground state geometries (S_0_). GaussSum 2.1 [55] was used for calculating the contribution of molecular orbitals (MO) in electronic transitions.

## 3. Results and discussion

**Scheme 1.**
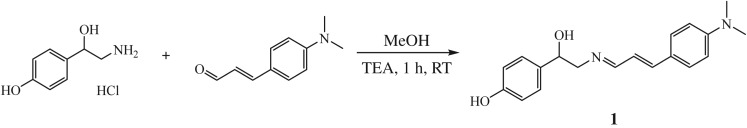
Synthesis of **1**.

Sensor **1** was synthesized by combination of octopamine and 4-dimethylaminocinnamaldehyde with 77.6% yield in MeOH (scheme 1). Compound **1** was characterized with analytic methods such as ESI mass analysis and ^1^H and ^13^C NMR.

### 3.1. Fluorescence ratiometric response of **1** toward Ag^+^

**Figure 1.**
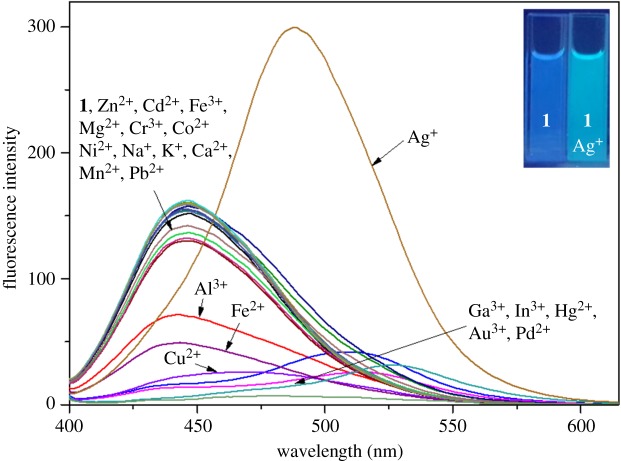
Fluorescence spectra of **1** (5 µM) with manifold metal ions (6 equiv).

To study the fluorometric sensing abilities of **1**, we conducted the selectivity experiment upon addition of 6 equiv of various metal ions such as Al^3+^, In^3+^, Zn^2+^, Ga^3+^, Cd^2+^, Fe^2+^, Fe^3+^, Cu^2+^, Mg^2+^, Cr^3+^, Ag^+^, Co^2+^, Hg^2+^, Ni^2+^, K^+^, Na^+^, Ca^2+^, Pb^2+^, Mn^2+^, Pd^2+^ and Au^3+^ (figure 1). Excitation at 382 nm caused a fluorescence emission of **1** at 449 nm. In the presence of different metal ions, Al^3+^ and Fe^2+^ decreased the fluorescence intensity of **1**, and Cu^2+^, Ga^3+^, In^3+^ and Hg^2+^ quenched it. Only Ag^+^ showed a bathochromic shift from 449 to 488 nm. There were no or little spectral changes with the other metal ions. The results demonstrated that sensor **1** could be used as a selective ratiometric fluorescence sensor for detection of Ag^+^. Importantly, the use of **1** as a ratiometric fluorescence sensor for Ag^+^ might be very meaningful, because only a few ratiometric fluorescence silver sensors have been reported to date [9,26,56–59].

**Figure 2.**
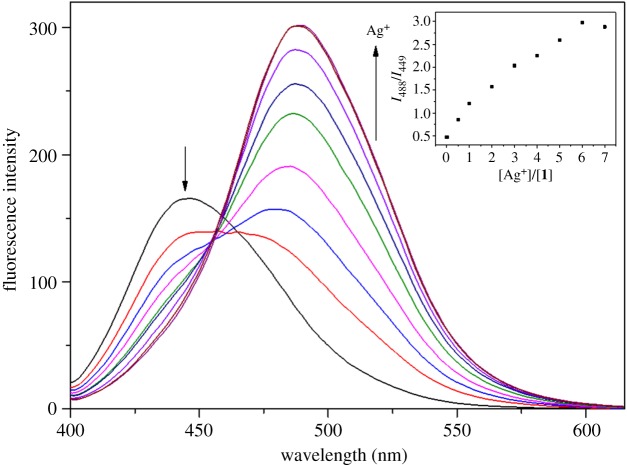
Fluorescence spectral changes of **1** (5 µM) with the addition of Ag^+^. Inset: plot of the fluorescence intensity at 488/449 nm versus the number of equiv of Ag^+^ added.

The fluorescence titration of **1** with Ag^+^ was carried out (figure 2). The fluorescence intensity steadily decreased at 449 nm and gradually increased at 488 nm up to 6 equiv of Ag^+^. To demonstrate the sensing property of **1** toward Ag^+^, we performed UV–visible titration (electronic supplementary material, figure S1). Upon the addition of Ag^+^, the absorbance at 350 nm dwindled and the band at 400 nm increased steadily up to 12 equiv of Ag^+^. An isosbestic point was apparent at 368 nm, which suggested that **1** and Ag^+^ constituted a single species.

For information on the binding mode of **1** with Ag^+^, Job plot and ESI mass spectrometry analyses were executed. Job plot [60] showed the 1 : 1 stoichiometric ratio (electronic supplementary material, figure S2). The positive-ion mass spectrum indicated the formation of **1** + Ag^+^ + 11 solvents [*m*/*z*: 727.32; calcd, 727.31] (electronic supplementary material, figure S3). The association constant of **1** with Ag^+^ was determined to be 7.00 × 10^4^ M^−1^ using Li's equation [61] based on the fluorescence titration data (electronic supplementary material, figure S4). The detection limit was calculated to be 1.49 µM (electronic supplementary material, figure S5) via 3*σ*/slope [62].

**Figure 3.**
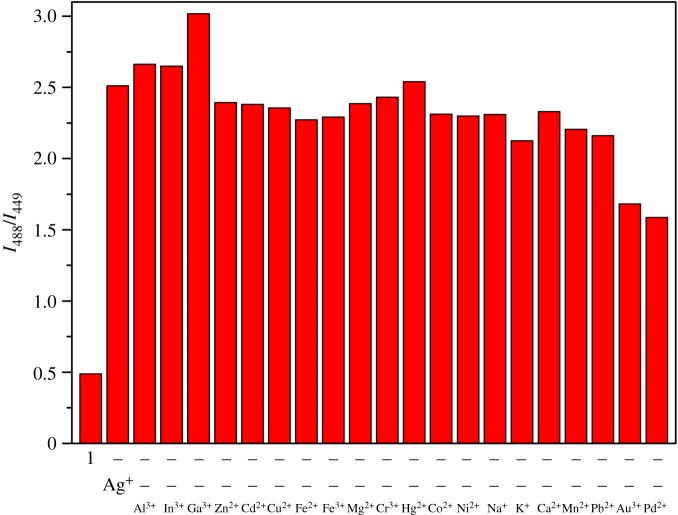
Fluorescence intensities (at 488/449 nm) of **1** (5 µM) with Ag^+^ (6 equiv) with various cations (6 equiv).

To ascertain the functional application prospect of **1**, the competition test was conducted in the presence of assorted metal ions such as Al^3+^, In^3+^, Zn^2+^, Ga^3+^, Cd^2+^, Cu^2+^, Mg^2+^, Fe^2+^, Fe^3+^, Cr^3+^, Ag^+^, Co^2+^, Hg^2+^, Ni^2+^, Na^+^, Mn^2+^, K^+^, Pb^2+^, Ca^2+^, Pd^2+^ and Au^3+^ (figure 3). Most metal ions did not show interferences to sense silver ion, and Pd^2+^ and Au^3+^ interfered about 30%. These results demonstrated that the sensor **1** was very selective for detection of Ag^+^.

**Figure 4.**
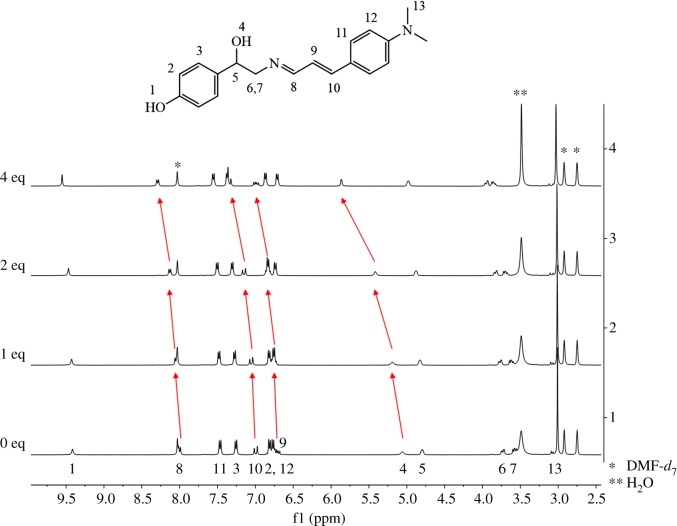
^1^H NMR titrations of **1** with the addition of Ag^+^ (0, 1, 2 and 4 equiv).

We implemented the ^1^H NMR titrations to further understand the binding properties of **1** toward Ag^+^ (figure 4). Upon addition of 4 equiv of Ag^+^ to **1**, most of the protons showed down-field shifts while proton H_12_ and H_13_ displayed no shift. In particular, protons H_4_, H_8_, H_9_ and H_10_ exhibited large down-field shifts. These chemical shifts indicated that the oxygen atom of the ethyl moiety and the nitrogen atom of the imine might associate with silver ion.

**Figure 5.**
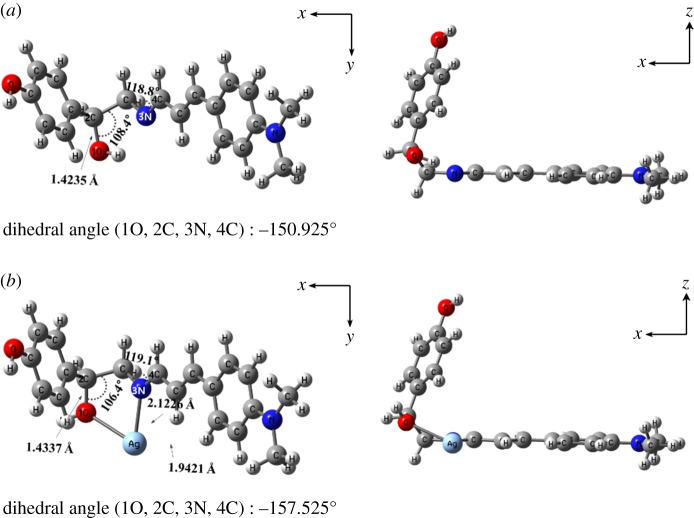
Energy-minimized proposed structures of (*a*) **1** and (*b*) **1**–Ag^+^ complex.

**Scheme 2.**
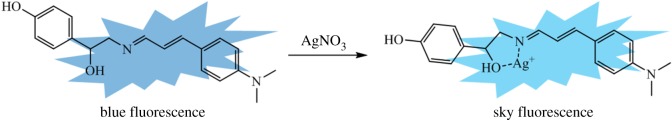
The proposed fluorescent sensing mechanism of Ag^+^ by **1**.

To further investigate the sensing mechanism of **1** with Ag^+^, the DFT and TD-DFT calculations were applied. First, we executed the geometric optimizations of **1** and **1**–Ag^+^ complex (figure 5). Ag atom was applied to the LANL2DZ/ECP, and the main atoms were employed to the B3LYP/6-31G(d,p) method. Energy-minimized structures of **1**–Ag^+^ and **1** species showed folded structures with the dihedral angles of 1O, 2C, 3N, 4C = −150.925° and −157.525°, respectively. Next, the electronic transitions of **1** and **1**–Ag^+^ complex were examined at the optimized geometries (S_0_). For **1**, the main MO contribution was calculated to be the HOMO → LUMO transition (372.52 nm, electronic supplementary material, figure S6), which was assigned to be the intramolecular charge transfer. For **1**–Ag^+^ complex, the main MO contribution was calculated to be the HOMO → LUMO transition (398.69 nm, electronic supplementary material, figure S7), which displayed the ligand-to-metal charge-transfer. These results showed that the binding of **1** with Ag^+^ caused a bathochromic shift (372.52 to 398.69 nm) and generated the shift of fluorescence intensity (449 to 488 nm) (electronic supplementary material, figure S8). Based on the analysis of Job plot, ESI mass, ^1^H NMR titrations and theoretical calculations, the sensing mechanism of **1** with Ag^+^ is described in scheme 2.

### 3.2. Fluorogenic and colorimetric responses of **1** toward S^2−^

**Figure 6.**
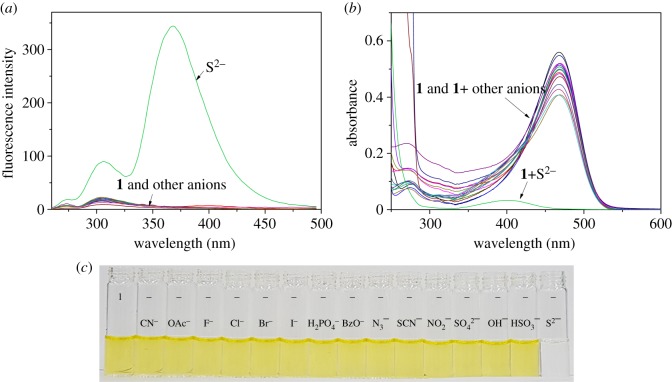
(*a*) Fluorescence and (*b*) absorption spectra of sensor **1** (10 µM) with different anions in buffer (bis-tris, pH = 7.0, 10 mM). (*c*) The colour changes of sensor **1** (10 µM) with various anions.

We examined the fluorescent and colorimetric sensing abilities of **1** in the presence of different anions such as Cl^−^, CN^−^, F^−^, Br^−^, I^−^, OAc^−^, H_2_PO_4_^−^, N_3_^−^, BzO^−^, SCN^−^, NO_2_^−^, SO_4_^2−^, OH^−^, HSO_3_^−^ and S^2−^ in bis-tris buffer (pH = 7.0, 10 mM, figure 6). The enhanced emission was shown only for sulfide with an excitation of 266 nm, whereas **1** and other anions exhibited no fluorescence emission (figure 6*a*). Also, there were no or little spectral changes for **1** and other anions (figure 6*b*), while **1** with S^2−^ showed a significant decrease of absorbance at 470 nm, which induced a colour change from yellow to colourless. This observation could be explained as follows. F^−^ with a strong basicity and hydrogen bonding character could form hydrogen bonding to excess water molecules instead of the deprotonation of any phenolic OH of sensor **1**. In contrast, S^2−^ with a strong basicity and less hydrogen bonding character might deprotonate more acidic phenolic proton of **1** instead of forming hydrogen bonds with less acidic water molecules, and then, showing colour change. These results suggested that sensor **1** had ability as a superior fluorogenic and colorimetric chemosensor for sulfide. Importantly, there are only a few sensors for the detection of S^2−^ with a dual method [32,63–65]. In addition, this is a first instance that a single chemosensor can recognize both silver(I) and sulfide, to the best of our knowledge.

**Figure 7.**
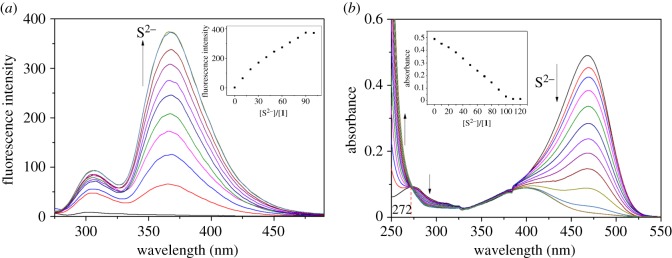
(*a*) Fluorescence and (*b*) absorption spectral changes of **1** (10 µM) with increasing concentrations of S^2−^. Inset: plot of (*a*) fluorescence at 368 nm and (*b*) absorbance at 470 nm versus the number of equiv of S^2−^ added.

We executed the fluorescence and UV–visible titrations of **1** with S^2−^ (figure 7). Upon the addition of S^2−^, the fluorescent intensity at 368 nm gradually increased up to 90 equiv of S^2−^ (figure 7*a*). In UV–visible titration (figure 7*b*), the absorbance at 260 nm increased, and the band at 470 nm steadily decreased with an isosbestic point at 272 nm. The tendency of dwindling absorbance at 470 nm was continued up to 110 equiv of S^2−^. These results demonstrated that **1** reacted with sulfide to form a single species.

To understand the interaction properties of **1** with S^2−^, we conducted Job plot and ESI mass spectrometry analyses. Job plot [60] showed the 1:1 stoichiometric ratio of **1** with S^2−^ (electronic supplementary material, figure S9). The negative-ion mass spectrum indicated the formation of **1** – H^+^ [*m/z*: 309.35; calcd, 309.16] (electronic supplementary material, figure S10). On the basis of fluorescence titration data, the association constant of **1** and sulfide was calculated to be 1.82 × 10^3^ M^−1^ using Li's equation [61] (electronic supplementary material, figure S11). The detection limit was determined to be 14.8 µM (electronic supplementary material, figure S12) by 3*σ*/slope [62].

**Figure 8.**
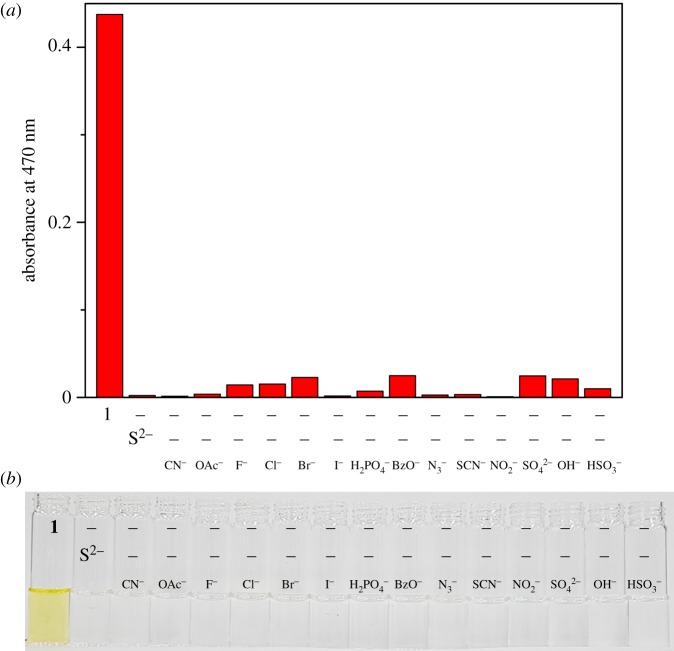
(*a*) UV–visible absorbance (at 470 nm) of **1** (10 µM) toward S^2−^ with various anions. (*b*) The colour changes of **1** (10 µM) with S^2−^ in the absence and presence of other anions.

We checked the practical abilities of **1** for sensing S^2−^ in the presence of other anions. For fluorescence recognition (electronic supplementary material, figure S13), CN^−^, Br^−^, BzO^−^, SO_4_^2−^ and OH^−^ displayed interferences of 30, 80, 55, 63, and 64%. However, CN^−^, BzO^−^, SO_4_^2−^ and OH^−^ still showed discernible fluorescence emission. For colorimetric recognition (figure 8), all of the anions showed no inhibition of S^2−^. These results explained that **1** could detect effectively S^2−^ without disturbance of other anions using two methods together.

For further information for practicality of **1**, we investigated the pH dependence of **1** for detection of sulfide. For fluorescence response (electronic supplementary material, figure S14*a*), sensor **1** could recognize the sulfide in pH range of 3 to 7. For colorimetric response (electronic supplementary material, figure S14*b*), sensor **1** could discern S^2−^ in pH range of 4 to 10. These observations suggested that S^2−^ could be well detected by **1** under both acid and base conditions by increasing fluorescence intensity and changing colour, respectively.

**Figure 9.**
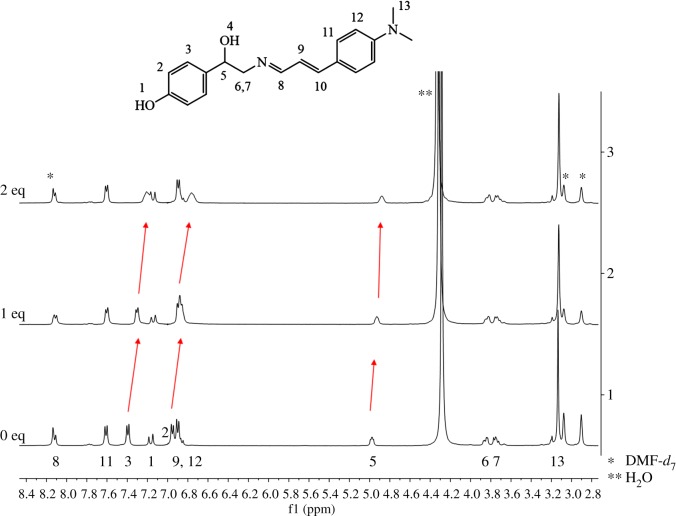
^1^H NMR titrations of **1** with the addition of S^2−^ (0, 1 and 2 equiv).

**Scheme 3.**
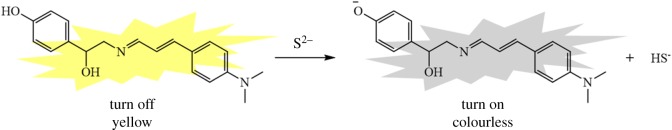
The proposed sensing mechanism of **1** toward sulfide.

^1^H NMR titrations were conducted to further examine the sensing mechanism of **1** with S^2−^ (figure 9). Hydroxyl protons were not observed due to the D_2_O solvent. As the concentration of sulfide increased, H_2_, H_3_ and H_5_ showed up-field shifts. There was no shift for other protons. These results suggested that the hydroxyl proton of the phenol group of **1** might be deprotonated by sulfide, inducing the fluorescence enhancement. Based on the analysis of Job plot, ESI mass and ^1^H NMR titration, we depict the sensing mechanism of **1** toward S^2−^ in scheme 3.

## 4. Conclusion

We designed a new multifunctional chemosensor **1** for detection of silver(I) and sulfide. Silver(I) with **1** showed the ratiometric fluorescence from blue to sky. **1** bound to Ag^+^ with a 1:1 ratio, which was determined by Job plot and ESI mass analyses. The limit of detection turned out to be 1.49 µM and there was no interference for sensing silver(I) by **1**. The sensing properties were understood via ^1^H NMR titrations and DFT calculations. In addition, sensor **1** recognized sulfide with an obvious fluorescence enhancement and colour change from yellow to colourless. The sensing mechanism of **1** with S^2−^ was studied through ESI mass, Job plot and ^1^H NMR titration. In particular, this is a first instance that a single chemosensor can recognize both silver(I) and sulfide, to the best of our knowledge.

## Supplementary Material

Supporting Information

## Data accessibility

All of the data in this study are included in figures and electronic supplementary material, and freely available.
