# Supplementary material for: A multi-functional chemosensor for highly selective ratiometric fluorescent detection of silver(I) ion and dual turn-on fluorescent and colorimetric detection of sulfide
Source: R Soc Open Sci. 2018 Jun 13;5(6):180293. doi: 10.1098/rsos.180293 (PMC6030272; doi:10.1098/rsos.180293)
Supplement: Supporting Information [file rsos180293supp1.docx]

**Supporting Information**

**A multi-functional chemosensor for highly selective ratiometric fluorescent detection of silver(I) ion and dual turn-on fluorescent and colorimetric detection of sulfide**

Ji Hye Kang, Ju Byeong Chae, Cheal Kim*

*Department of Fine Chem., Seoul National Univ. of Sci. and Tech., Seoul 139-741, Korea. Fax: +82-2-973-9140; Tel: +82-2-970-6681; E-mail:* [*chealkim@seoultech.ac.kr*](mailto:chealkim@seoultech.ac.kr).

**
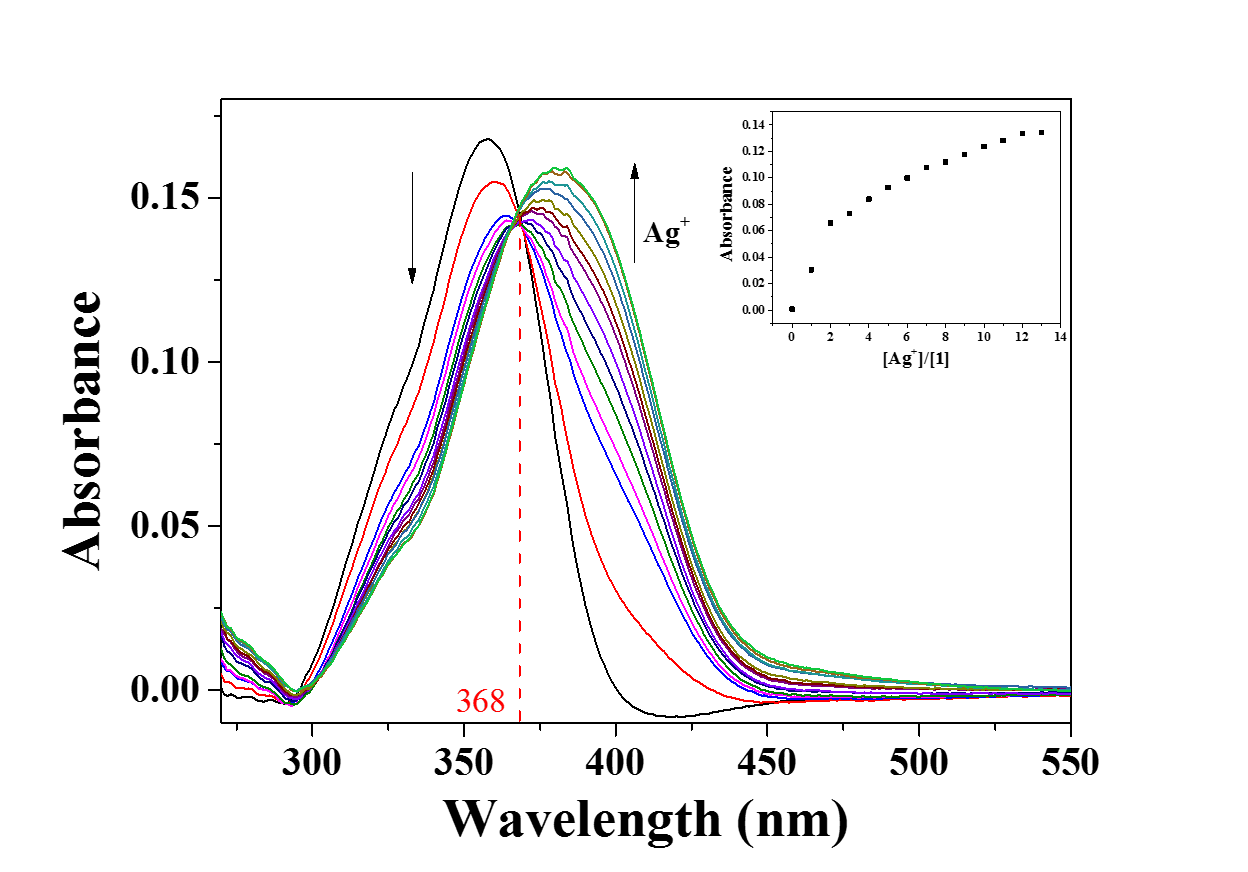
**

**Fig. S1.** Absorption spectral changes of **1** (5 μM) upon the addition of Ag^+^. Inset: Plot of the absorbance at 400 nm versus the number of equiv of Ag^+^ added.

**Fig. S2.** Job plot for binding ratio of **1** with Ag^+^. The total concentration of **1** with Ag^+^ was 30 μM.


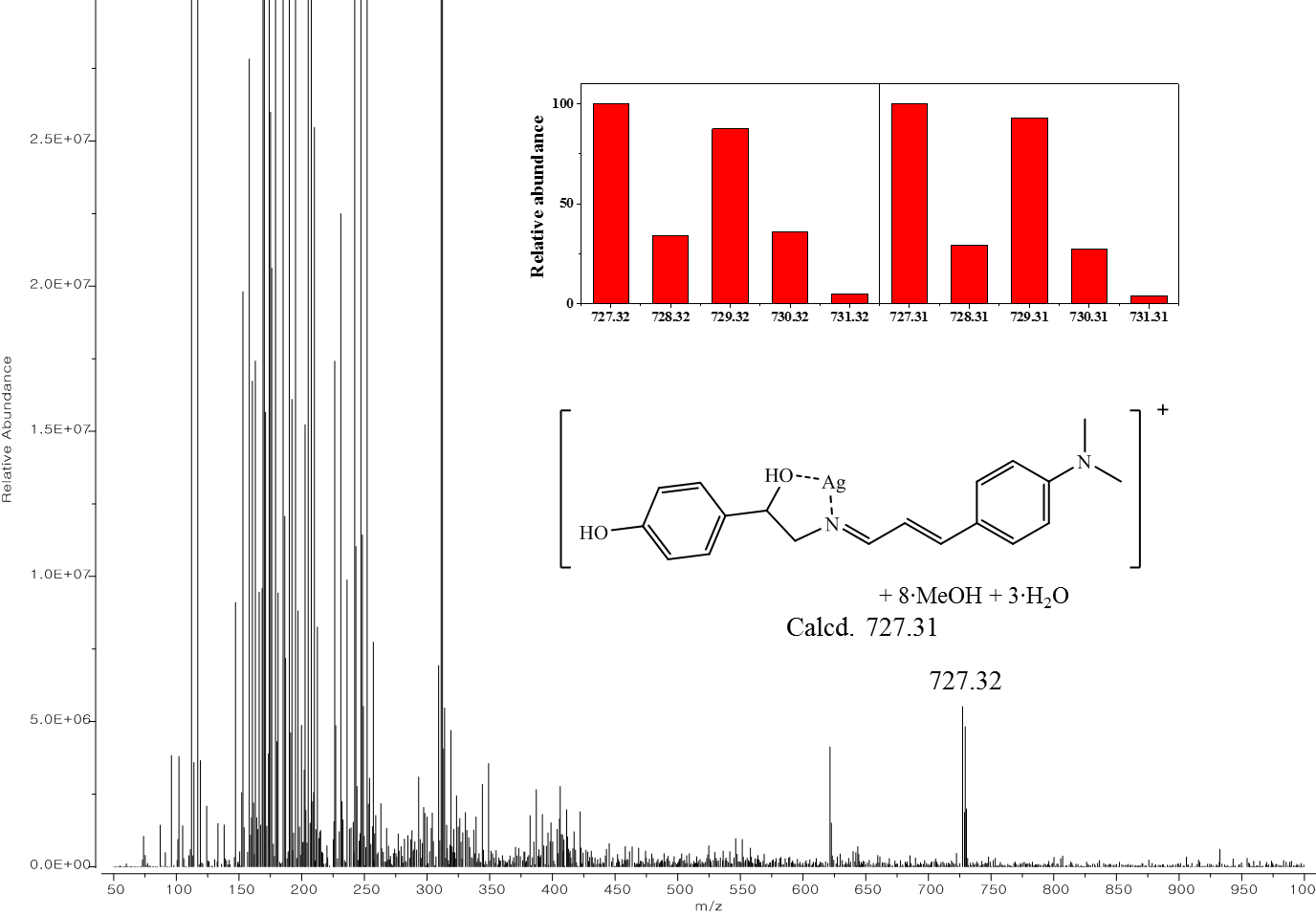


**Fig. S3.** Positive-ion electrospray ionization mass spectrum of **1** (100 μM) upon addition of Ag^+^ (1 equiv).

**Fig. S4.** The association constant of **1** toward Ag^+^ by using the Li’s equation based on fluorescence titration at 488/449 nm.

**Fig. S5.** Determination of the detection limit of **1** (5 μM) for Ag^+^ based on change of intensity at 488/449 nm.

(a)

(b)

**Fig. S6.** (a) The theoretical excitation energies and the experimental UV-vis spectrum of **1**. (b) The major electronic transition energy and molecular orbital contributions for **1** (H = HOMO and L = LUMO).

(a)

(b)

**Fig. S7.** (a) The theoretical excitation energies and the experimental UV-vis spectrum of **1**-Ag^+^ complex. (b) The major electronic transition energies and molecular orbital contributions of **1**-Ag^+^ complex (H = HOMO and L = LUMO).


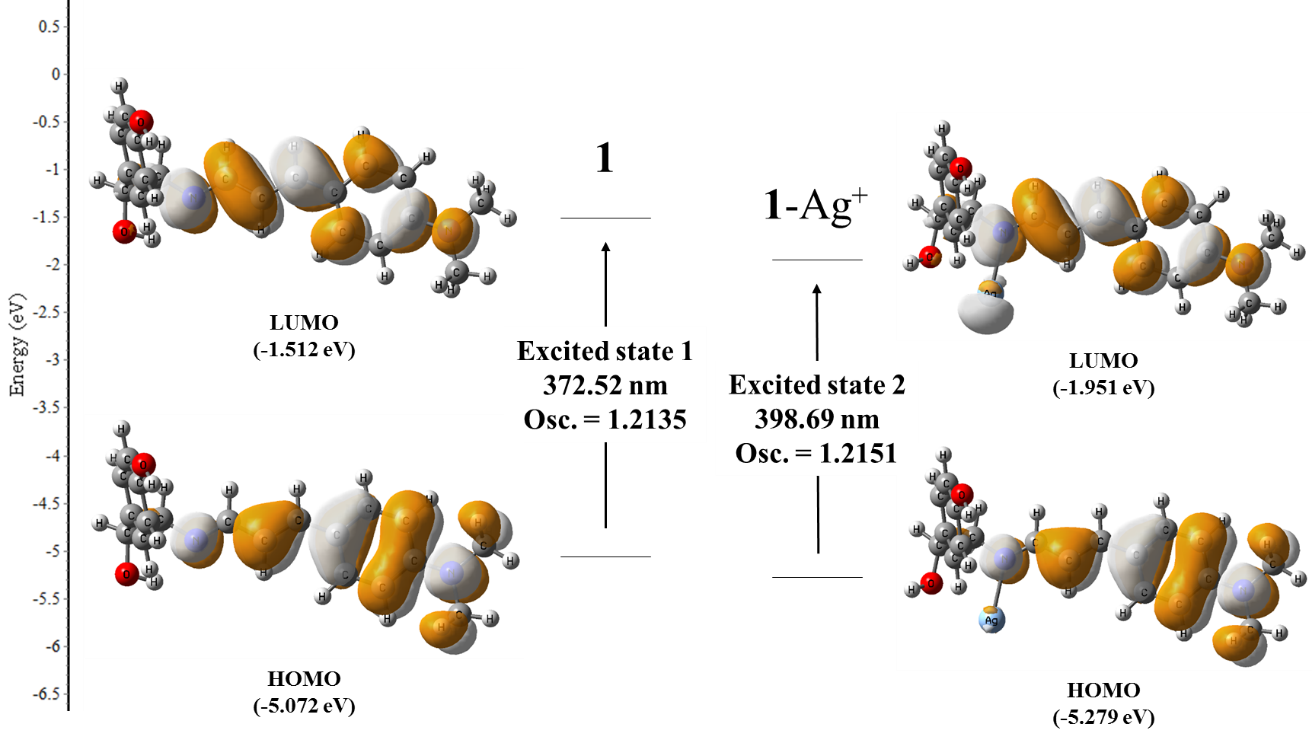


**Fig. S8.** Molecular orbital diagrams of **1** and **1**-Ag^+^ complex using TD-DFT methods.

**Fig. S9.** Job plot for association ratio of **1** with S^2-^. The total concentration of **1** with S^2-^ was 70 μM.


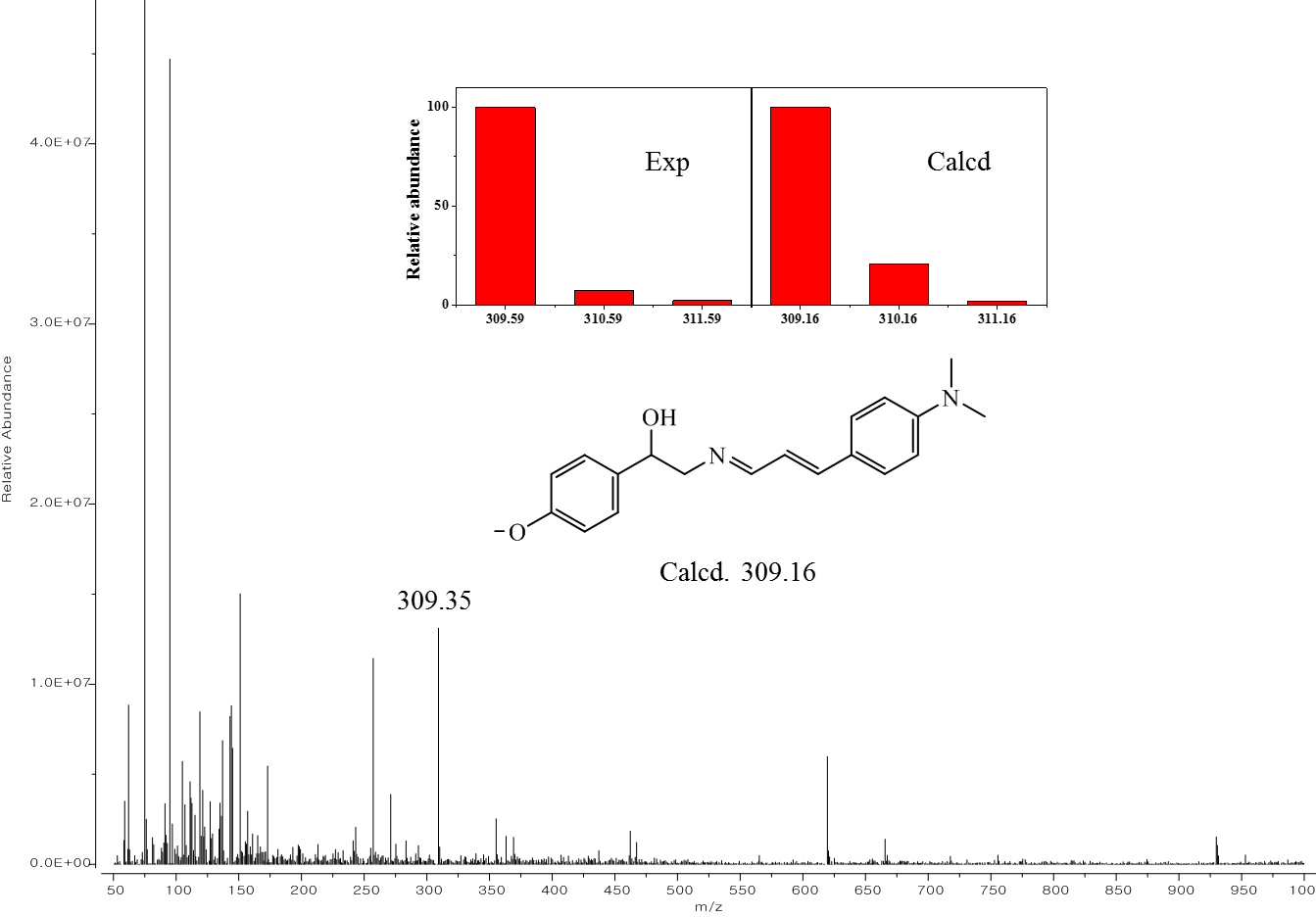


**Fig. S10.** Negative-ion electrospray ionization mass spectrum of **1** (100 μM) upon addition of S^2-^ (1 equiv).

**Fig. S11.** The association constant of **1** with S^2-^ by using the Li’s equation based on fluorescence titration at 405 nm.

**Fig. S12.** Determination of the detection limit of **1** (10 μM) for S^2-^ based on change of intensity at 368 nm.

**Fig. S13.** Fluorescence intensities (at 368 nm) of **1** (10 μM) toward S^2-^ with various anions.

(a)

(b)

**Fig. S14.** (a) Fluorescence intensities (at 368 nm) and (b) absorbance of **1** (10 μM) and **1**-S^2-^, respectively, at pH range of 2-12.
